# Comparison of Bioengineered Scaffolds for the Induction of Osteochondrogenic Differentiation of Human Adipose-Derived Stem Cells

**DOI:** 10.3390/bioengineering11090920

**Published:** 2024-09-14

**Authors:** Elena Fiorelli, Maria Giovanna Scioli, Sonia Terriaca, Arsalan Ul Haq, Gabriele Storti, Marta Madaghiele, Valeria Palumbo, Ermal Pashaj, Fabio De Matteis, Diego Ribuffo, Valerio Cervelli, Augusto Orlandi

**Affiliations:** 1 Anatomic Pathology Section, Department of Biomedicine and Prevention, University of Rome Tor Vergata, 00133 Rome, Italy; elena.fiorelli1@gmail.com (E.F.); terriacasonia093@gmail.com (S.T.); orlandi@uniroma2.it (A.O.); 2 Plastic Surgery Unit, Department of Surgery “Pietro Valdoni”, Sapienza University of Rome, 00133 Rome, Italy; diego.ribuffo@uniroma1.it; 3 Department of Industrial Engineering, University of Rome Tor Vergata, 00133 Rome, Italy; arsalan.ulhaq@iit.it (A.U.H.); fabio.dematteis@roma2.infn.it (F.D.M.); 4 Interdepartmental Research Centre for Regenerative Medicine (CIMER), University of Rome Tor Vergata, 00133 Rome, Italy; 5 Department of Plastic Surgery, University of Rome Tor Vergata, 00133 Rome, Italy; gabriele.stortimd@gmail.com (G.S.); valeriocervelli@virgilio.it (V.C.); 6 Department of Experimental Medicine, University of Salento, 73100 Lecce, Italy; marta.madaghiele@unisalento.it; 7 Department of Experimental Medicine, University of Rome Tor Vergata, 00133 Rome, Italy; valeria.palumbo.25@students.uniroma2.eu; 8 Department of Surgical Sciences, Catholic University Our Lady of Good Counsel, 1005 Tirana, Albania; e.pashaj@unizkm.al; 9 Department of Biomedical Sciences, Catholic University Our Lady of Good Counsel, 1005 Tirana, Albania

**Keywords:** regenerative medicine, tissue engineering, scaffolds, biomaterials, osteochondral differentiation, human adipose-derived stem cells

## Abstract

Osteochondral lesions may be due to trauma or congenital conditions. In both cases, therapy is limited because of the difficulty of tissue repair. Tissue engineering is a promising approach that relies on designed scaffolds with variable mechanical attributes to favor cell attachment and differentiation. Human adipose-derived stem cells (hASCs) are a very promising cell source in regenerative medicine with osteochondrogenic potential. Based on the assumption that stiffness influences cell commitment, we investigated three different scaffolds: a semisynthetic animal-derived GelMA hydrogel, a combined scaffold made of rigid PEGDA coated with a thin GelMA layer and a decellularized plant-based scaffold. We investigated the role of different biomechanical stimulations in the scaffold-induced osteochondral differentiation of hASCs. We demonstrated that all scaffolds support cell viability and spontaneous osteochondral differentiation without any exogenous factors. In particular, we observed mainly osteogenic commitment in higher stiffness microenvironments, as in the plant-based one, whereas in a dense and softer matrix, such as in GelMA hydrogel or GelMA-coated-PEGDA scaffold, chondrogenesis prevailed. We can induce a specific cell commitment by combining hASCs and scaffolds with particular mechanical attributes. However, in vivo studies are needed to fully elucidate the regenerative process and to eventually suggest it as a potential approach for regenerative medicine.

## 1. Introduction

Damaged tissues and organ failure are major problems in modern medicine. Osteochondral lesions can occur after a traumatic event or due to congenital and/or pathophysiological conditions that can affect both younger and older populations [1]. Those injuries are common in weightbearing joints and diagnosis may be delayed if the damage is subtle [2]. Nowadays, clinical treatments for osteochondral lesion are reconstructive and orthopedic surgery can partially repair the original tissue function [3]. 

Tissue engineering is an uprising field whose main goal is to provide tissue regeneration, combining cells from the body with a scaffold [4,5]. Scaffolds essentially provide an ideal 3D environment for cells to receive biochemical and mechanical stimuli close to their native tissue microenvironment pushing them to regenerate a tissue or organ [6]. Scaffolds must be biocompatible, support adhesion, interaction, migration, proliferation of cells and new matrix deposition. Furthermore, the scaffold must elicit a minimal immune response after implantation to avoid in vivo rejection [5]. Materials used for scaffolds can influence the commitment of mesenchymal stem cells and mechanical properties, like elastic modulus, play a role in this process [7,8]. Among the various biomaterials, methacrylated gelatin (GelMA), a naturally derived semi-synthetic biopolymer, is emerging as a potential candidate to sustain tissue repair due to its low toxicity and immunogenicity, cell–material interaction and its metalloproteinase sensibility. GelMA is a photocrosslinkable bioink with tunable properties [9,10]. GelMA has been tested as a good chondrocyte support as well as matrix formation support [11]. On the other hand, GelMA, like many other natural biopolymers, demonstrates poor mechanical properties such as mechanical strength, and stiffness [12]. Those limitations can be overcome by blending GelMA with synthetic polymers such as polyethylene glycol diacrylate (PEGDA). The latter, despite having less biocompatibility to GelMA, by its printability, can produce composite and versatile scaffolds with specific biomechanical properties with broad applicability, including osteochondrogenesis [13,14]. Most recently, tissue engineering has focused its attention on scaffolds made from decellularized vegetables and plants. The latter are cost effective and easily available as well as modifiable and exhibit good biomechanical properties [15]. Plant-based scaffolds have been reported in cartilage and bone regeneration [16]. Moreover, celery-based scaffolds offer promising advantages such as availability all year long, biocompatibility, stiffness, porosity, biodegradability and cost-effectiveness [17]. Finally, the hydrogels (i.e., GelMA, PEGDA) as well as plant-based scaffolds have been reported to not elicit inflammatory responses and to be suitable for in vivo transplants [18,19]. So, in this study, we tested different combinations of biopolymers to develop an ideal scaffold with specific biomechanical properties to support osteochondral differentiation of seeded hASCs. The latter are easy to harvest and are available in large quantities, offering a feasible alternative to other mesenchymal stem cells [20]. Human ASC differentiation capabilities as well as broad immunomodulatory and anti-inflammatory properties make them a promising cell resource for regenerative medicine purposes [21]. 

## 2. Materials and Methods

Different scaffolds were fabricated and characterized, as summarized in Table 1 and detailed below.

### 2.1. Synthesis of GelMA

GelMA was synthesized by following a previous protocol [22]. In brief, 10 g type A gelatin from porcine skin (gel strength 300 g Bloom, Merck, Darmstadt, Germany) was dissolved in 100 mL phosphate-buffered saline (PBS, Merck) at 60 °C to obtain 10% *w*/*v* gelatin solution while stirring vigorously. The solution was cooled down to 50 °C, and 8 mL methacrylic anhydride (MA; Merck) at a rate of 0.5 mL/min was added to this solution, to create photo-responsive methacrylate groups on gelatin chains (Supplementary Figure S1A). The solution was then diluted by adding 300 mL warm PBS to stop further methacrylation. The diluted solution was dialyzed using dialysis tubes with a 12–14 kDa molecular weight cut off, in distilled water changed twice a day for a week at 40 °C to remove residual impurities. Finally, the solution was freeze-dried for 3 days and stored at −80 °C until further use.

### 2.2. ^1^H-NMR Spectroscopy

The degree of substitution (DoS) of GelMA was calculated using nuclear magnetic resonance (NMR) spectroscopy (Bruker Avance 700 MHz spectrometer equipped with a Triple resonance TXI probe and a SampleXpress Lite autosampler, Billerica, MA, USA), according to published protocols [23]. In particular, the DoS, from the NMR spectra, indicates how many amines and -OH groups in gelatin the methacryloyl groups were substituted by during the synthesis process (Supplementary Figure S1B).

### 2.3. GelMA Hydrogel Fabrication

Hydrogels were prepared by radical crosslinking of GelMA with a photo-initiator (PI, Irgacure 2959, CIBA chemicals, Basel, Switzerland). Firstly, 0.1% *w*/*v* of PI was added to 10 mL PBS in the dark and placed for 1 h at 80 °C in a hot water bath to dissolve the PI. After, 10–15% *w*/*v* freeze-dried GelMA was added to the solution, and left to dissolve for another 30 min in the same hot water bath. Successively, 40 μL of GelMA solution was pipetted out and placed on a sterile glass slide between two spacers of 0.8 mm thickness with another sterile glass slide placed on top and placed under the UV source (wavelength 365 nm) for 1 min at room temperature. Circular hydrogels of 6 mm diameter were finally obtained (Supplementary Figure S1C). For hASC loading, before UV crosslinking, the GelMA solution was cooled down to 37 °C. Cells were added to the solution at a density of 300,000 cells/40 µL and pipetted out on a sterile glass slide between the two spacers. After UV crosslinking, hydrogels were washed in PBS and placed in a low adherence plate with Dulbecco’s Modified Eagle Medium (DMEM, Merck) plus 10% fetal bovine serum (DMEM-FBS 10%) and then kept in the incubator (Supplementary Figure S2A).

### 2.4. PEGDA-GelMA Scaffold Fabrication

The 3D-printed PEGDA scaffolds were fabricated using the projection micro-stereolithography technique [24]. In brief, the precursor solution for 3D printing was a blend of polyethylene glycol diacrylate (MW 575 Da PEGDA, Merck) as the photo-sensitive polymer, curcumin extracted from curcuma longa as the light absorbing dye and Irgacure 819 (Merck) as PI. Irgacure 819 and curcumin were dissolved in PEGDA:ethanol (*v*/*v* 3:1 solution) and left overnight under stirring in a dark environment to obtain the precursor solution for 3D printing. The intended geometry of the scaffold was first created in the AutoCAD. A stereolithographic (.stl) file was then exported and was used to virtually cut the 3D geometry into 2D projections using the Slic3r software version 1.3.0 (https://slic3r.org/). An overhead projector (Acer X1385WH, New Taipei City, Taiwan) containing a high-pressure mercury arc lamp with a luminous flux of 3400 lumens was used to expose 2D projections onto the precursor solution in which a 3-axis stage was already submerged to 100 µm. The precursor solution exposed with a first projection for 6 sec rapidly underwent crosslinking via free radical photo-polymerization reaction. The first printed 100 µm thick layer was submerged again for 100 µm in a fresh precursor solution and exposed again with the second projection, to print the second layer on top of the first one. Stacking of those 2D layers resulted in a 3D scaffold, in which curcumin prevented light from penetrating too deep into the photo-sensitive working printing process [24]. Finally, a thin layer of 0.5% and 5% GelMA (15 µL) was added and crosslinked (Supplementary Figure S1D). For hASC loading, before UV crosslinking of the GelMA solution, cells were added at a density of 300,000 cells/15 µL and pipetted out on 3D-printed PEGDA scaffolds (as previously described). After UV crosslinking, scaffolds were washed in PBS and placed in a low adherence plate with DMEM-FBS 10% and kept in the incubator (Supplementary Figure S2B).

### 2.5. Preparation of Plant-Based Scaffolds

Celery from local market was sliced longitudinally or transversally with a mandolin with a thickness of 0.5 mm and a punch was then used to cut it in a round shape with a diameter of 0.4 mm. Celery was decellularized adding 1% sterile sodium dodecyl sulfate (SDS; Merck) in water for either 24 h or 72 h on the shaker at room temperature. After washing three times with PBS and 70% ethanol solution for 24 h, scaffolds were left in 70% ethanol until use [15] (Supplementary Figure S1E). For hASC loading, scaffolds were first dried at RT for the evaporation of ethanol and then soaked in the culture medium for 1 h before cell seeding. Successively, scaffolds were placed in a low adherence plate without medium, and cells were seeded at a density of 200,000 cells/15 μL of medium per scaffold as reported. After 30 min in the incubator to allow cells to adhere, DMEM-FBS 10% was added then kept in the incubator (Supplementary Figure S2C).

### 2.6. Decellularization Assessment

The assessment of the decellularization was made by encapsulating the scaffolds in paraffin and sectioning along the entire length, sections were then stained with hematoxylin for 3 min and eosin for 30 sec, once mounted with coverslips, the stainings were checked for cells presence and photographed by an E600 Eclipse microscope with Dxm1200F digital camera with ACT-1 software 9.2 (Nikon, Tokyo, Japan) [15].

### 2.7. Ultrastructural Analysis

Scanning electron microscopy (SEM) using the Zeiss LEO 420 scanning electron microscope (Carl Zeiss, Oberkochen, Germany) was performed as reported [25]. Briefly, samples were fixed with 10% buffered formalin and post-fixed with osmium tetroxide (OsO_4_; Merck) at 4 °C for 60 min. Successively, samples were dehydrated and finally put in the critical point machine and placed with silver paint on the stub.

### 2.8. Biomechanical Tests

The mechanical properties of scaffolds were investigated by means of unconfined compression tests. Scaffolds (*n* = 6–7 each type of scaffold) were mounted on a Z1.0 TH testing machine (Zwick Roell, Ulm, Germany), equipped with a 10-Newton load cell and a bath chamber. Hydrated samples were then tested at RT at a displacement rate of 0.01 mm/s up to 50% deformation. The slope of the linear elastic region at low strain values (in the 0–5% range) was calculated to estimate the compressive elastic modulus.

### 2.9. Assessment of Scaffold Degradability

The degradability of GelMA hydrogels over time was assessed by incubating the gels in a collagenase solution (0.5 mg/mL, 125 CDU/mg) in Hank’s Balanced Salt Solution containing 3 mM CaCl_2_ at 37 °C [10]. Sample weight was assessed at 0, 15, 30, 45 min and 1 h. Since PEGDA-based and plant-based scaffolds are not degraded by collagenase, for both those scaffolds the degradability was evaluated by incubating them in different solutions: distilled water, 1 M NaOH and 1 M HCl at 37 °C. The samples were then weighed over 8 weeks for plant-based and 48 h for PEGDA respectively, and mass loss was calculated as reported [10].

### 2.10. Assessment of Swelling Degree

Swelling tests for scaffolds were performed using a conventional gravimetric method [10]. Briefly, scaffolds were placed into a tube with 1.0 mL deionized water at room temperature and the weight was measured, at different time points, with a precision balance. In particular, GelMA hydrogels and PEGDA scaffolds were weighed every 5 min, whereas plant-based scaffolds were weighed every 15 min. For the latter, the swelling test was performed on bigger samples than usual to allow the weighing.

### 2.11. Human Adipose-Derived Stem Cells (hASC) Isolation and Cell Culture

Human adipose-derived stem cells (hASCs) were isolated from subcutaneous fat samples derived from randomly selected donors who underwent liposuction procedures [25].

Adipose tissue was centrifuged at 3000 rpm for 5 min to separate the isolate the aqueous phase and treat it with DMEM-collagenase 0.1% and let it rest at 37 °C for 45 min. Once fully digested, the aqueous phase was centrifuged at 1200 rpm for 5 min. After filtering, the pellet was seeded in a petri dish with warm medium DMEM-FBS 10%. The study was approved by the local ethics committee (Protocol no. 0019144, study n. 172.18 approved on 31 January 2019), and all patients signed the informed consent form.

### 2.12. Viability Assay

Following the protocol provided by the kit manufacturer, cell-seeded scaffolds were placed in a 96-well plate with 100 µL of DMEM-FBS 10% with 10 µL of Cell Counting Kit 8 solution (CCK8; Merck). Scaffolds were incubated for 3 h in the incubator (37 °C, 5% CO_2_). A volume of 50 µL from the CCK8 medium for each well was pipetted in a 96-well plate; it was placed in a plate reader and absorbance at 450 nm was measured (Promega, Madison, WI, USA).

### 2.13. Dead/Live Staining

Dead/live staining (Biotium, Fremont, CA, USA) is based on fluorescence probes calcein and ethidium bromide. Briefly, samples were incubated for 45 min with 4 µM calcein and 2 µM ethidium bromide, washed with PBS and visualized with confocal microscopy.

### 2.14. Osteochondrogenic Differentiation Assay 

The 3D GelMA hydrogels were fixed in buffered formalin 10%, dehydrated and embedded in paraffin. Samples were sliced and colored with Alizarin red for 3 min, the excessive staining was washed, then they were dehydrated in acetone. Finally, they were treated in xylene and mounted. Similarly, samples for Alcian blue staining were submerged with a different solution as the protocol suggested (Bio Optica, Milan, Italy), washed with distilled water, dehydrated with alcohol and xylene and mounted.

### 2.15. Immunofluorescence and Confocal Imaging

For immunofluorescence, samples were fixed with 4% paraformaldehyde and then permeabilized with a solution of PBS + 0.2% Tween (Thermo Fisher Scientific, Waltham, MA, USA) for 5 min. Scaffolds were stained as reported [26]. Briefly, samples were incubated with rabbit polyclonal anti-collagen type II alpha 1 (COL2A1) antibody (Merck) and rabbit polyclonal anti-osteocalcin (OCN) antibody (Thermo Fisher Scientific) for 1 h at room temperature. Then, after washing in PBS, they were incubated for 30 min with a specific goat anti-rabbit secondary antibody (1:50 Thermo Fisher Scientific). Hoechst solution (1:1500, Merck) was used for nuclear counterstain. Images were captured with FV1000 Fluoview confocal microscope (Olympus, Tokyo, Japan).

### 2.16. Statistical Analysis

Experiments were performed in triplicate and the number of samples was 3–7 for each condition. Results are expressed as mean ± standard error of mean (SEM). Significant differences between sets of data were analyzed for statistical significance using analysis of variance (ANOVA) and *t* tests; differences were considered significant for *p* value < 0.05. 

## 3. Results

### 3.1. GelMA Hydrogel Fabrication and Mechanical Properties

**Figure 1:**
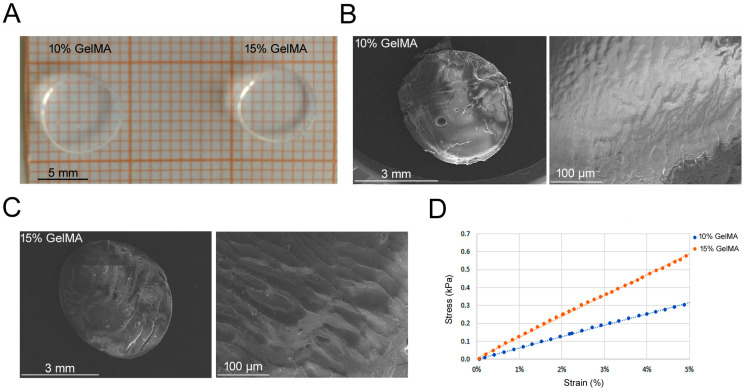
GelMA scaffold characterization and biomechanical test. (**A**) Hydrogels made with 10% and 15% of GelMA. (**B**,**C**) SEM imaging of 10% and 15% GelMA hydrogel, respectively. (**D**) Stress and strain graph showing a representative elastic modulus for 10% and 15% GelMA hydrogels.

Tuning mechanical properties such as elastic modulus has been reported to affect stem cell commitment [8,23]. As shown in the Supplementary Figure S1B, NMR spectra evidenced a methacrylation degree of 88% for liquid GelMA. The five protons in phenylalanine aromatic rings around 7.2 ppm were used for normalization. The signal from the multiple peaks around 2.9 ppm were used to calculate the DoS [27]. After DoS assessment, 10% and 15% GelMA hydrogel (Figure 1A) ultrastructures were visualized by SEM. Both 10% and 15% GelMA hydrogels showed a similar dense and compact surface with no visible pores (Figure 1B,C). As concerning the biomechanical properties of GelMA hydrogels, the elastic modulus for 10% GelMA was 5.94 ± 0.7 kPa, whereas for 15% GelMA it was higher, specifically 11.91 ± 2.0 kPa (*p* < 0.05; representative trends in Figure 1D).

### 3.2. PEGDA-GelMA Scaffold Characterization and Mechanical Properties

**Figure 2:**
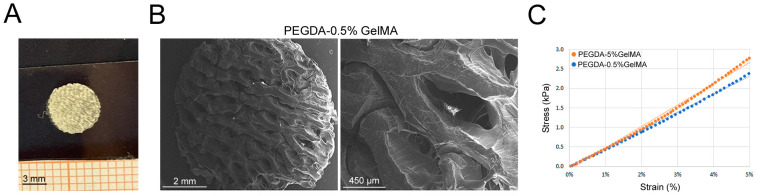
PEGDA scaffold production. (**A**) The final PEGDA scaffold with GelMA coating. (**B**) SEM images of PEGDA scaffold (GelMA 0.5%) showing its ultrastructure and porosity. (**C**) Stress and strain graph showing a representative elastic modulus for PEGDA-0,5% GelMA and PEGDA-5% GelMA.

We also tested the scaffold made of PEGDA, known to be a biomaterial with a high stiffness (more rigid) [13]. Since PEGDA does not allow cell adhesion by itself for the absence of binding sites, we overcame the problem by using a light GelMA coating, thus producing a PEGDA-GelMA hybrid scaffold. PEGDA was printed as a porous scaffold and filled with 0.5% GelMA or 5% GelMA, without cells (Supplementary Figure S1D and Figure 2A,B). The analysis of PEGDA-0.5% GelMA scaffold ultrastructure by SEM (Figure 2B) showed a porous surface constituted of 800 µm wide pores. The compressive stiffness of the two hybrid scaffolds was higher compared to GelMA; however, there was not a statistical difference between PEGDA-0.5% GelMA and PEGDA-5% GelMA, with elastic moduli of 40.3 ± 6.7 kPa and 42.5 ± 10.6 kPa, respectively (Figure 2C). However, based on less variability in observed stiffness values of PEGDA-0.5% GelMA, we decided to proceed with the latter for the following tests.

### 3.3. Plant-Based Scaffold Characterization and Mechanical Properties

**Figure 3:**
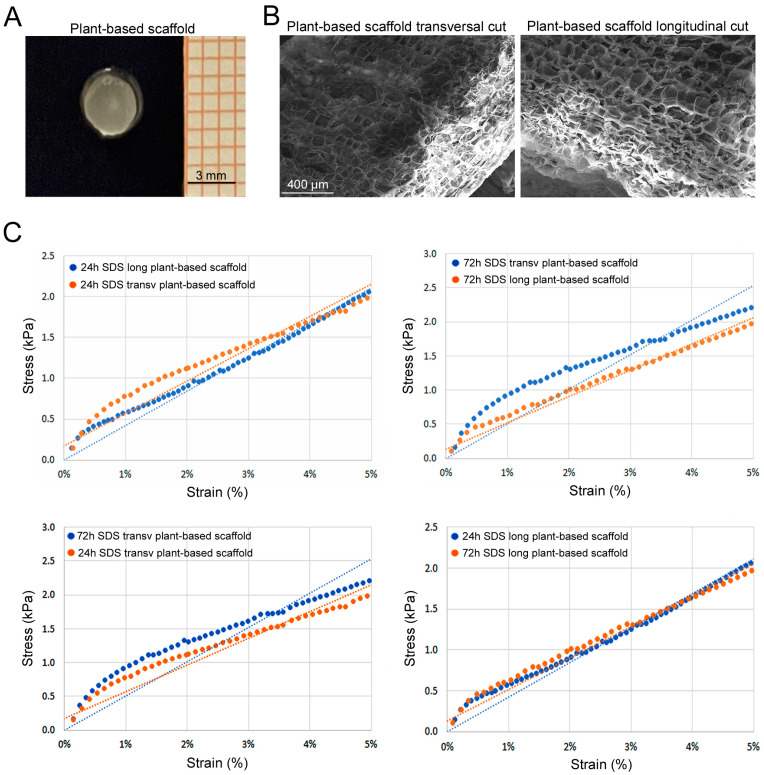
(**A**) Celery-based scaffold after decellularization and sterilization in 70% ethanol. (**B**) SEM images of decellularized celery-based scaffolds (24 h and 72 h SDS protocol). (**C**) Representative trends of stress and strain (0–5% strain) comparing different decellularized protocols (24 h and 72 h) and cut orientation (longitudinally cut and transversal cut).

We produced plant-based scaffolds (Supplementary Figure S1E and Figure 3A). Histological analysis of hematoxylin-and-eosin-stained sections revealed a successful decellularization of cellulose structure with both 24 and 72 h SDS incubation times (Supplementary Figure S3). SEM ultrastructure analysis of celery-based scaffolds evidenced the characteristic pore size of around 150 µm for both transversal and longitudinal cuts (Figure 3B). Interestingly, celery-based scaffolds showed an average stiffness comparable to PEGDA scaffolds (Figure 3C). In particular, celery-based scaffolds obtained with the 24 h SDS decellularization protocol showed an elastic modulus of 46.76 ± 8.43 kPa for slices cut transversally, and 42.51 ± 7.78 kPa for longitudinal cut ones. Similar values were obtained for celery samples treated with the 72 h SDS protocol. In fact, longitudinal cut samples showed an elastic modulus of 43.08 ± 10.03 kPa, while the transversal cut ones had a modulus of 47.64 ± 6.40 kPa. Altogether, there was not a statistical difference either for the cut orientation or for the SDS protocol. Since the slightly higher stiffness of the transversal cut samples, we decided to proceed with the latter (24 h SDS protocol) for the biological tests.

### 3.4. Degradation Tests

**Figure 4:**
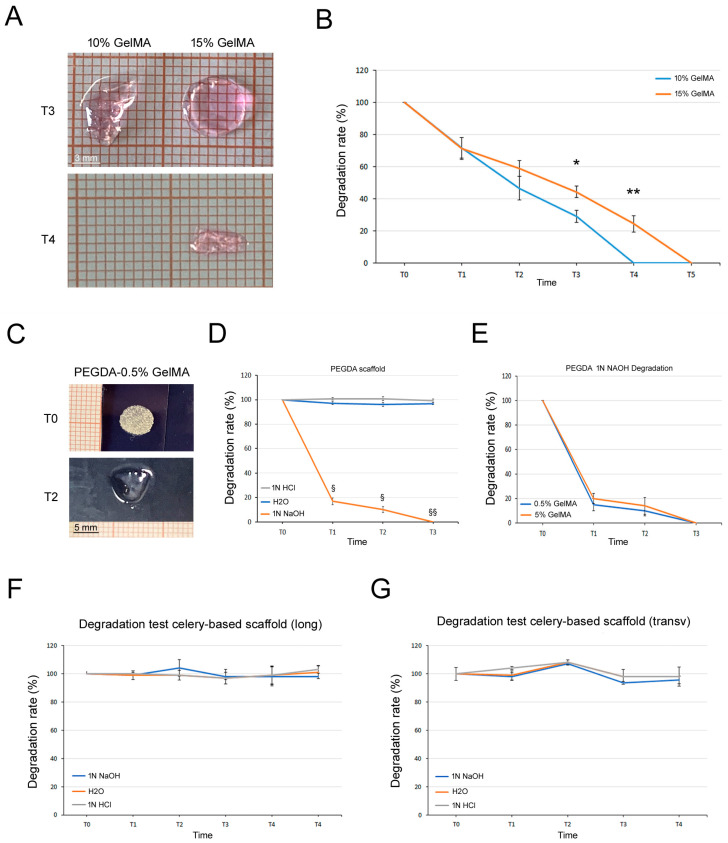
Degradation tests. (**A**) Representative images of 10% and 15% GelMA hydrogel degradation with significant differences at T3 and T4. (**B**) Graph showing the degradation trend of 10% and 15% GelMA hydrogel in 0.5% collagenase at different points (T, 15 min intervals). (**C**) Representative images of PEGDA-0.5% GelMA scaffold degradation at T0 (baseline) and T2 (20 h of incubation). (**D**) Graphs showing the degradation trend of PEGDA scaffold in H2O, 1N NAOH and 1N HCL, and (**E**) the comparison between PEGDA-0.5% GelMA and PEGDA-5% GelMA in 1N NAOH. The different time points are: T0 (baseline), T1 (18 h of incubation), T2 (20 h of incubation) and T3 (44 h of incubation). (**F**,**G**) Degradation test graphs of celery-based scaffolds, (**F**) cut longitudinally or (**G**) transversally, incubated with H2O, 1N NAOH and 1N HCL, respectively. T0, T1, T2, T3 and T4 stands for baseline, 2, 4, 6 and 8 weeks. Results are reported as mean ± SEM of *n* = 3 samples/groups. T test: * *p* < 0.05 and ** *p* < 0.001; § *p* < 0.0001 and §§ *p* < 0.00001.

Considering their different nature, a specific degradation test was performed for each type of scaffold. For GelMA hydrogels, the degradability was tested incubating them in PBS-0.5% collagenase type I. As expected, 10% GelMA hydrogels were fully degraded after 45 min (T4, *p* < 0.001 vs. 15% GelMA), meanwhile the more dense 15% GelMA hydrogels were fully digested after 90 min (T5; Figure 4A,B). Since PEGDA is not degradable with collagenase [28], we used different pH solutions. As shown in Figure 4C–E, PEGDA scaffolds fully degraded only at the basic pH condition (1N NAOH) with a similar trend for PEGDA-0.5% and PEGDA-5% GelMA. We also treated celery-based scaffolds (both longitudinal and transversal cuts, 24 h SDS protocol) with different pH solutions without registered any changes in weight over 2 months of time (Figure 4F,G), in accordance with the literature [29].

### 3.5. Swelling Tests

**Figure 5:**
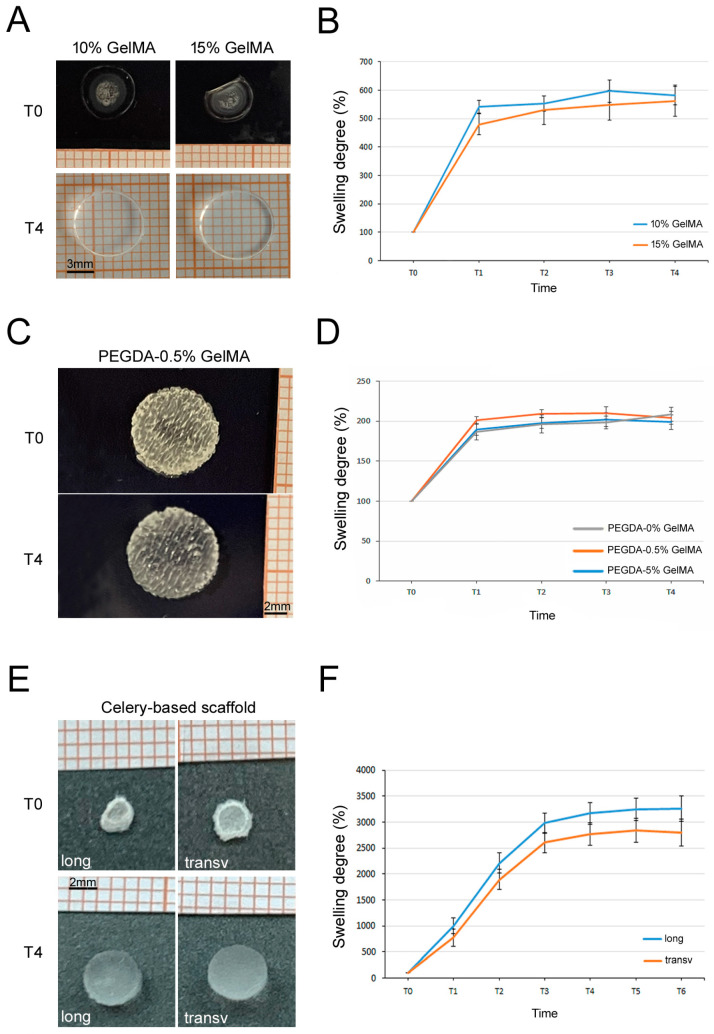
Swelling tests. (**A**) Representative images of 10% and 15% GelMA hydrogel before and after the swelling test at T0 (baseline) and T4 (20 min). (**B**) Graph showing the swelling trend of 10% and 15% GelMA hydrogel, each time point (T) is a 5 min interval. (**C**) Representative images of PEGDA-0.5% GelMA scaffold before and after the swelling degree at T0 (baseline) and T4 (20 min). (**D**) Graph showing the swelling trend of PEGDA-0.5% GelMA, PEGDA-5% GelMA and PEGDA-0% GelMA scaffold for each time point (T, 5 min intervals). (**E**) Representative images of celery-based scaffolds cut longitudinally and transversally dried (T0, baseline) and hydrated (T4, 40 min). (**F**) Graph showing the swelling trend of celery-based scaffolds cut longitudinally or transversally at different time points (T, 10 min-intervals). Since the standard size of scaffold was too light and did not weigh enough to permit a precise measurement, a bigger scaffold was made purposely to perform this test. Results are reported as mean ± SEM of *n* = 3 samples/group.

As previously mentioned, GelMA is made of gelatin, a very hydrophilic component with a quick water uptake after 5 min (Figure 5A,B). After that time both hydrogels remained stable with no substantial differences. PEGDA-GelMA scaffolds also adsorbed water after 5 min, with also a passive trapping inside the structure, remaining unaltered over time (Figure 5C,D). We performed the test even without the GelMA coating, observing no impact on the absorption of the structure since it was a negligible amount (Figure 5D). The high porosity of the celery-based scaffold with its hydrophilic cellulose structure allowed an extreme water absorption as well as its holding (Figure 5E,F). The weight rapidly increased up both for the longitudinal and transversal cut scaffolds, with no significant differences in swelling between the longitudinal and the transversal cut. Because of the similar biomechanical properties showed, we decided to proceed with the transversal cut scaffold only for the slightly higher stiffness (Figure 5F).

### 3.6. Scaffold-Dependent hASC Survival and Differentiation

**Figure 6:**
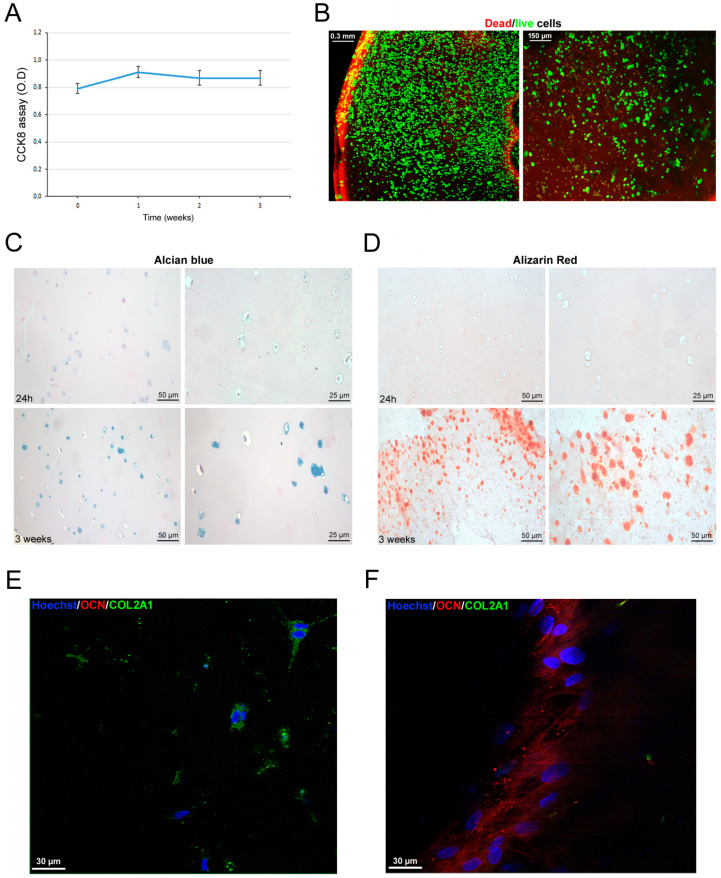
hASC survival and differentiation in GelMA hydrogels. (**A**) CCK8 assay of hASCs encapsulated in the GelMA hydrogel at different time points (1 week intervals). Results are reported as mean ± SEM of *n* = 3 samples/group. (**B**) Representative confocal imaging of Dead/live fluorescence staining of hASCs in the GelMA hydrogel after 3 weeks of culture. Red cells are dead while green cells are alive. (**C**) Alcian blue staining of GelMA hydrogel after 24 h and 3 weeks of culture. (**D**) Alizarin red staining of GelMA hydrogel after 24 h and 3 weeks of culture. (**E**,**F**) Confocal imaging of immunofluorescence for COL2A1 (green) and OCN (red) in hASCs encapsulated in GelMA hydrogel after 3 weeks of culture.

**Figure 7:**
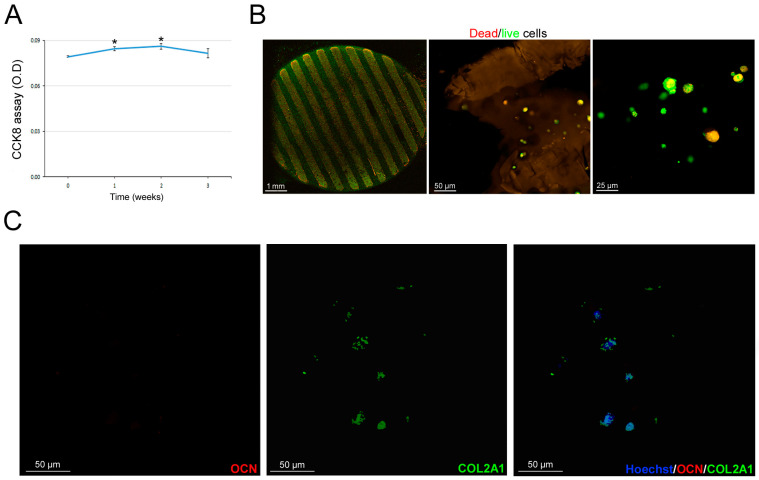
hASC survival and differentiation in PEGDA scaffold. (**A**) CCK8 assay of hASCs seeded in the PEGDA scaffold at different time points (1 week intervals). Results are reported as mean ± SEM of *n* = 3 samples/group. T test: * *p* < 0.05. (**B**) Representative confocal imaging of Dead/live fluorescence staining of hASCs seeded in the PEGDA scaffold after 3 weeks of culture. (**C**) Confocal imaging of immunofluorescence for COL2A1 (green) and OCN (red) in hASCs seeded in the PEGDA scaffold after 3 weeks of culture.

**Figure 8:**
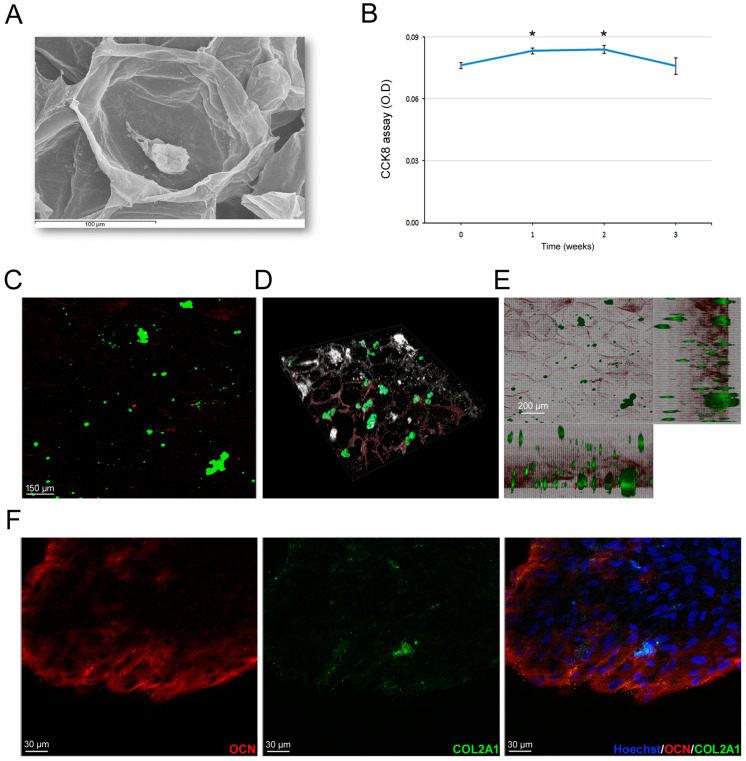
hASC survival and differentiation in celery-based scaffold. (**A**) SEM imaging of a single hASC inside a niche of the celery-based scaffold. (**B**) CCK8 assay of hASCs seeded in the celery-based scaffold at different time points (1 week intervals). Results are reported as mean ± SEM of *n* = 3 samples/group. T test: * *p* < 0.05. (**C**) Representative confocal imaging of Dead/live fluorescence staining of hASCs seeded in the scaffold after 3 weeks of culture. (**D**) Confocal 3D stack and (**E**) the projection of hASC distribution inside the scaffold. (**F**) Confocal imaging of immunofluorescence for COL2A1 (green) and OCN (red) in hASCs seeded in the PEGDA scaffold after 3 weeks of culture.

Once we selected the most promising scaffolds, we proceeded with the hASC-related biological tests. Preliminarily, we excluded the 15% GelMA hydrogel because of evident suffering signs from cell proliferation and survival assays in our preliminary experiences, likely due to its more dense structure. So, we proceeded with 10% GelMA hydrogels and investigated hASC survival inside the hydrogel over time. In particular, at 3 weeks’ time after culture, hASCs viability remained stable (Figure 6A). That finding was confirmed by the Dead/live staining (Figure 6B). Furthermore, we investigated hASC differentiation commitment inside the GelMA hydrogels. Microscopic analyses of histochemical stainings revealed that hASC were positive for GAG (Figure 6C) and calcium deposition (Figure 6D) after 3 weeks of culture, supporting the spontaneous dual chondrogenic and osteogenic commitment of hASCs in 3D culture [25]. Those findings were confirmed by the expression of the chondrogenic marker COL2A1 (green signal, Figure 6E) and of osteogenic marker OCN (red signal, Figure 6F), indicating that neither commitment specifically prevails. The cell stability inside the GelMA hydrogels indicated that differentiated hASCs did not increase in number with a physiological cell turnover. Regarding PEGDA-GelMA scaffolds, cells seeded inside the scaffolds slightly increased their viability during the first and second week of culture (*p* < 0.05, Figure 7A); successively, cell viability returned to the initial condition. The Dead/live staining confirmed cell viability at 3 weeks with a physiological cell turnover (Figure 7B). In parallel, at 3 weeks, confocal images documented the presence of COL2A1, supporting a spontaneous tendency toward cartilage differentiation. Human ASCs seeded on celery-based scaffold (Figure 8A) showed a slight but significant increase in cell viability at week 1 and 2; successively, they returned to the initial value (*p* < 0.05, Figure 8B). The Dead/live staining confirmed cell viability at 3 weeks with a physiological cell turnover (Figure 8C), similarly to the PEGDA scaffold. Interestingly, confocal imaging, documented a regular spatial distribution of alive hASCs (green) inside the celery-based scaffold (Figure 8D,E). Moreover, immunofluorescence revealed the predominant expression of OCN, supporting a spontaneous tendency toward osteogenic differentiation (Figure 8F).

## 4. Discussion

It has been reported that stiffness influences cell commitment [7,8]. We relied on the assumption that due to different mechanical scaffold properties, cells can be directed to a cell lineage. hASCs have shown to be able commit towards different lineages according to the mechanical environment around them, with rigid stimuli cells more likely to progress toward bone commitment, while with softer stimuli cells tend to go toward a chondrogenic commitment [8,23,30]. The aim of this study was to test how scaffolds with different biomaterials and mechanical properties can influence hASC osteochondrogenic differentiation. We chose three types of biomaterials, GelMA, PEGDA and celery, each of them with distinct compositions, geometries and mechanical properties that permitted us to create diverse microenvironments. The advantages of GelMA are due to its biocompatibility, degradability, biomechanical tunability, biological properties and adaptability to 3D bioprinting [30,31]. In this light, our mechanical tests proved that GelMA has tunable mechanical properties [32]. In fact, we confirmed, by compressive stress–strain curves, that higher GelMA concentration resulted in higher scaffold stiffness. The average hydrogel stiffness was around 6 kPa for 10% GelMA and around 12 kPa for 15% GelMA. However, it should be noted that during 10% and 15% GelMA hydrogel production, some limitations were encountered with higher concentrations of GelMA. In particular, a higher temperature was required to keep the 15% of GelMA viscous solution sufficiently liquid for easy pipetting. In addition, in our preliminary data, hASCs encapsulation in 15% GelMA hydrogel induced cell suffering with low cell viability. Because of its cytotoxicity and production problems due to its viscosity, we excluded GelMA at high concentrations for the following tests. So, we chose 10% GelMA for the in vitro studies and biological tests. Concerning the degradation degree, both hydrogels (10% GelMA and 15% GelMA) rapidly dissolved in the enzyme solution; however, the higher density of 15% GelMA justified the slight delay in degradation compared with 10% GelMA. Further experiments are needed to verify this finding and clarify the degradation rate overtime in a physiological condition. That aspect is crucial for tissue regeneration strategies because, from a biological standpoint, scaffolds provide initial support for cell survival and differentiation, then scaffold degradation provides the necessary space for the newly formed tissues. In any case, the degradation can be modified by using methyl acrylate addition during GelMA production [27]. Regarding the swelling degree, the hydrogel incorporated a similar amount of water, where both 10% and 15% GelMA hydrogels showed a rapid water uptake as well as holding. From the biological perspective, CCK8 data of hASCs-seeded GelMA showed only a slight increase in cell viability after the first week of culture, then it remains stable until three weeks. We hypothesized that, once seeded, cells rapidly undergo differentiation and stop proliferating with a stable turnover. The Dead/live assay at three weeks confirmed this finding, showing mainly living cells and rare dead cells, supporting the existence of a slow physiological cell turnover. In some experiences, we evaluated the timeline until 4 weeks but did not observe any significant differences compared to 3 weeks. In addition, we demonstrated that GelMA hydrogels support both chondrogenic and osteogenic differentiation of hASCs, as shown by histochemical and immunofluorescence stainings at three weeks. In fact, hASCs encapsulated in GelMA hydrogels accumulated both GAGs as well as calcium deposits. Moreover, encapsulated hASCs expressed both chondrogenic and osteogenic markers COL2A1 and OCN, respectively. The osteochondral differentiation occurring at the same time is not an unexpected finding. In fact, we previously demonstrated the presence of two different CD146+ and CD146- hASCs subpopulations that display specific intrinsic characteristics with a different spontaneous chondrogenic and osteogenic commitment, respectively [25]. Moreover, we could exclude chondrocyte hypertrophy, since hASCs are already negative for hypertrophic markers during chondrogenic commitment [25]. Both chondrogenic and osteogenic commitments were reported for mesenchymal cells cultured in GelMA hydrogels [24,33,34]; however, it is a common practice to add exogenous differentiating factors not considering the intrinsic and spontaneous cell commitment as well as the effect of the extracellular microenvironment itself. 

Due to the biomechanical characteristics of hydrogels, rigidity represents an important limit to overcome for their clinical use. To this aim, we produced a hybrid scaffold combining GelMA with PEGDA. The latter was used just to obtain a rigid porous scaffold to give a more solid and stiffer support to cells. In the literature, PEGDA has been often mixed in the liquid state with other bio-inks, including GelMA, to develop a stiffer structure to create the best chondrogenic and osteogenic conditions [35,36,37]. In those studies, authors produced a hybrid scaffold PEGDA-GelMA, mixing their liquid forms together and employed cells that were already committed (osteosarcoma, osteoblasts) or pharmacologically differentiated by exogenous factors (hASCs). Moreover, in some cases, cells were just seeded on the top of scaffolds, thus not really mimicking a 3D condition. In our experience, when we crosslinked PEGDA and GelMA together in various liquid proportions, the survival of hASCs was very low. That was probably due either to the harsh, poor permeable environment and/or to their nature. We decided to change approach and use the PEGDA scaffold itself, with the purpose of giving a stiffer structure, taking advantage of its great printability and mechanical properties, with a successively GelMA coating to permit cell attachment. Mechanical tests of PEGDA-0.5% GelMA showed a similar stiffness compared with PEGDA-5% GelMA, with PEGDA as the main rigid component. In particular, PEGDA-0.5% GelMA showed less variability among samples. Degradation tests showed that only 1N NAOH was capable of completely dissolving PEGDA, without any differences between 0.5% and 5% GelMA. In the swelling test, the PEGDA-GelMA scaffold showed to uptake and hold water in a similar and stable manner, with no variations associated to different GelMA percentages; however part of the retained water is likely due to a passive trapping in the porous structure. Regarding cell survival within the selected PEGDA-0.5% GelMA, the CCK8 assay showed a stable viability except a slight but significant increase in the initial phase. The stable viability was confirmed by the Dead/live staining that showed a physiological turnover at 3 weeks of culture. The turnover likely coincided with the initial cell commitment to chondrogenesis, as demonstrated by a predominant and specific COL2A1 expression. That finding is likely explained by the encapsulation of hASCs in a very low density hydrogel (0.5% GelMA), favoring chondrogenic differentiation [38,39]. So, in this case, there is no influence by the PEGDA structure but only GelMA microenvironment influence. However, hydrogels (i.e GelMA, PEGDA) have been reported to not elicit inflammatory responses and to be suitable for in vivo transplant [18,40]; the limitations of PEGDA and GelMA scaffolds are related to the fact that they are both chemically modified products and also animal-derived. Those aspects could create important safety issues for their future clinical applications [19]. The use of natural scaffolds, such as plant-based scaffolds, can overcome those limitations. Therefore, we decided to include in our study a celery-based scaffold. Interestingly, ultrastructural analysis and mechanical tests evidenced no significant difference in the decellularization process at 24 or 72 h of SDS protocol. Mechanical analysis of plant-based scaffolds showed the highest stiffness, especially the transversal cut one, more than PEGDA and GelMA. In general, the plant-based scaffolds had very high degrees of swelling, similarly to the GelMA hydrogel. Celery-based scaffolds were not degradable, as expected, and their cellulose-based non-degradable nature could offer a stable mechanical support in those areas where other types of scaffolds can collapse, also avoiding the release of toxic compounds. Those are all aspects that should be considered for their use in tissue engineering, especially in osteogenic regeneration. For the biological tests, we decided to use the transversal cut with 24 h SDS protocol that showed the higher stiffness. As concerning cell viability, the CCK8 assay showed a stable viability trend with a slight but significant increase in the initial phase, similarly to PEGDA-GelMA scaffolds. Data from Dead/live staining supported the existence of a physiological cell turnover. We hypothesize that, apart from an initial increase in cell number, hASCs stop growing and start osteogenic differentiation, as confirmed by the confocal fluorescent images showing predominant expression of OCN. Consistently with the assumption that the stiffness can address cell commitment, hASC differentiation at 3 weeks toward an osteogenic commitment likely reflects the high stiffness and porosity of some plant-based scaffolds, in line with findings from the literature [41],44]. Unlike PEGDA, cells are in close contact with the scaffold and directly influenced by its stiffness. The presence of some cells expressing COL2A1 is explained by the use of the heterogeneous unsorted hASC population [25].

Altogether, our scaffolds support the hypothesis that hASCs are able to differentiate accordingly to the different microenvironment stimuli and the appropriate scaffold without the use of exogenous differentiating factors. Plant-based scaffolds provided the best biomechanical stimuli to promote osteogenic commitment, while GelMA hydrogels supported both chondrogenic and osteogenic commitments accordingly to its concentration. In particular, when GelMA was used at lower concentration, it favored chondrogenesis, whereas at higher concentration, GelMA also stimulated osteogenic commitment. So, the concentration of GelMA can be crucial to influence cell commitment; however, higher concentrations can affect cell viability. Our findings derived from a time-limited in vitro study and our bioconstructs are limited to small osteochondral defects. So, long-term in vivo tests are needed to assess survival, vascularization and stability of the newly formed tissues. 

## 5. Conclusions

The use of specific scaffolds represents a valid support for tissue regeneration. We highlighted the role of the biomaterial structure and microenvironment to support cell survival, and to induce a spontaneous and specific cell commitment. The non-use of exogenous factors makes that approach safe for potential clinical trials. Higher stiffness sustained the tendency to osteogenic commitment. Higher stiffness of plant-based scaffolds favored osteogenesis, whereas low-density GelMA favored chondrogenesis. The next step will be the combined use of a specific sorted cell population, with intrinsic characteristics, with the best performing scaffold to optimize a particular commitment. Further studies are required to confirm in vivo the regeneration and maintenance of neoformed tissues, by vascularization process, in an extended timeframe.

## Figures and Tables

**Table 1:** Designed bioengineered scaffolds.

Scaffold Type	Fabrication Method
GelMA 10%	UV crosslinking
GelMA 15%	UV crosslinking
PEGDA + GelMA 0.5%	3D-printed PEGDA scaffolds with UV crosslinked 0.5% GelMA
PEGDA + GelMA 5%	3D-printed PEGDA scaffolds with UV crosslinked 5% GelMA
Celery 24 h transv	Celery sections sliced transversally and decellularized for 24 h
Celery 24 h long	Celery sections sliced longitudinally and decellularized for 24 h
Celery 72 h transv	Celery sections sliced transversally and decellularized for 72 h
Celery 72 h long	Celery sections sliced longitudinally and decellularized for 72 h

## Data Availability

Data are contained within the article or in the Supplementary Material.

## Supplementary Materials

The following supporting information can be downloaded at https://www.mdpi.com/article/10.3390/bioengineering11090920/s1, Figure S1: Scaffold synthesis and hydrogel production. (A) The chemical reaction between gelatin and methacrylate anhydride to produce GelMA. (B) Nuclear magnetic resonance spectra of gelatin and GelMA, confirming the substitution of primary amine groups by methacryloyl groups in GelMA. (C) Hydrogel production scheme, 40 µL of GelMA solution (containing 0.1% of the photo-initiator Irgacure 2959) are pipetted out on a coverslip between two spacers and exposed to UV for a minute. (D) Projection micro-stereolithography is used to print PEGDA scaffold. Successively, a droplet (15 µL) of GelMA solution (0.5% and 5%) is added, and the scaffold is exposed to UV light for 1 min. (E) Celery is cut and sliced into thin sections. The slices are decellularized by 1% SDS solution for 24 h or 72 h, washed in PBS and sterilized in 70% ethanol before cell culturing.; Figure S2: Scaffold cell seeding. (A) Cell seeding process, hASC suspension (300,000 cells/15 µL solution) is mixed to 10% GelMA solution and placed on a sterile cover slip that is exposed to UV light for 1 min for photo-crosslinking. Finally, GelMA hydrogel is placed in a non-adherent 24 multi-well plate and cultured with DMEM plus 10% FBS and kept in the incubator. (B) Encapsulation process of hASCs in PEGDA scaffold. A drop of 0.5% GelMA solution containing hASCs (300,000 cells/15 µL solution) is pipetted out on PEGDA scaffold and exposed to 1 min to UV light. Finally, the obtained scaffolds are placed in a non-adherent 24 multi-well plate and cultured with DMEM plus 10% FBS and kept in the incubator. (C) Cell seeding in celery-based scaffolds (transversal cut, 24 h SDS protocol). After making and sterilizing the scaffold, a drop of hASC suspension (200,000 cells/15 µL medium) is pipetted out on the top and let it rest for 2 h in the incubator. Subsequently, scaffolds are placed in a non-adherent 24 multi-well plate and cultured with DMEM plus 10% FBS.; Figure S3: Plant base decellularization. (A) Plant-based scaffold transversal cut decellularized after 24 h SDS protocol and (B) plant-based scaffold longitudinal cut decellularized after 24 h SDS protocol.
