# Supplementary material for: Comparison of Bioengineered Scaffolds for the Induction of Osteochondrogenic Differentiation of Human Adipose-Derived Stem Cells
Source: Bioengineering (Basel). 2024 Sep 14;11(9):920. doi: 10.3390/bioengineering11090920 (PMC11429422; doi:10.3390/bioengineering11090920)
Supplement: Supplementary file 1 [file bioengineering-11-00920-s001.zip › bioengineering-3140166-supplementary.pdf]

bioengineering

SUPPLEMENTAL INFORMATION

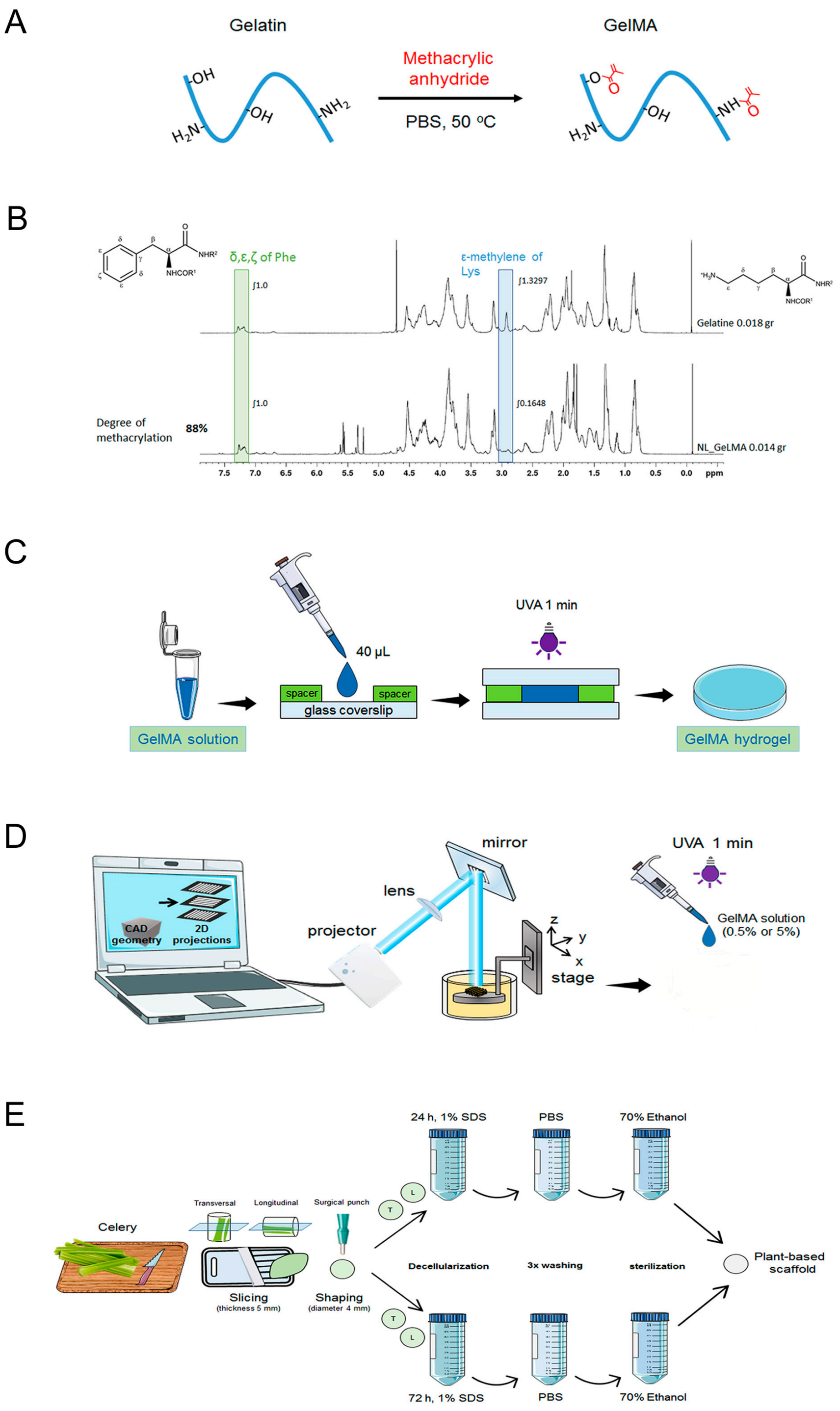

Figure S1. Scaffold synthesis and hydrogel production. (A) The chemical reaction between gelatin and methacrylate anhydride to produce GelMA. (B) Nuclear magnetic resonance spectra of gelatin and GelMA, confirming the substitution of primary amine groups by methacryloyl groups in GelMA. (C) Hydrogel production scheme, 40 µl of GelMA solution (containing 0.1% of the photo-initiator Irgacure 2959) are pipetted out on a coverslip between two spacers and exposed to UV for a minute. (D) Projection micro-stereolithography is used to print PEGDA scaffold. Successively, a droplet (15µL) of GelMA solution (0.5% and 5%) is added, and the scaffold is exposed to UV light

for 1 min. (E) Celery is cut and sliced into thin sections. The slices are decellularized by 1% SDS solution for 24 h or 72 h, washed in PBS and sterilized in 70% ethanol before cell culturing.

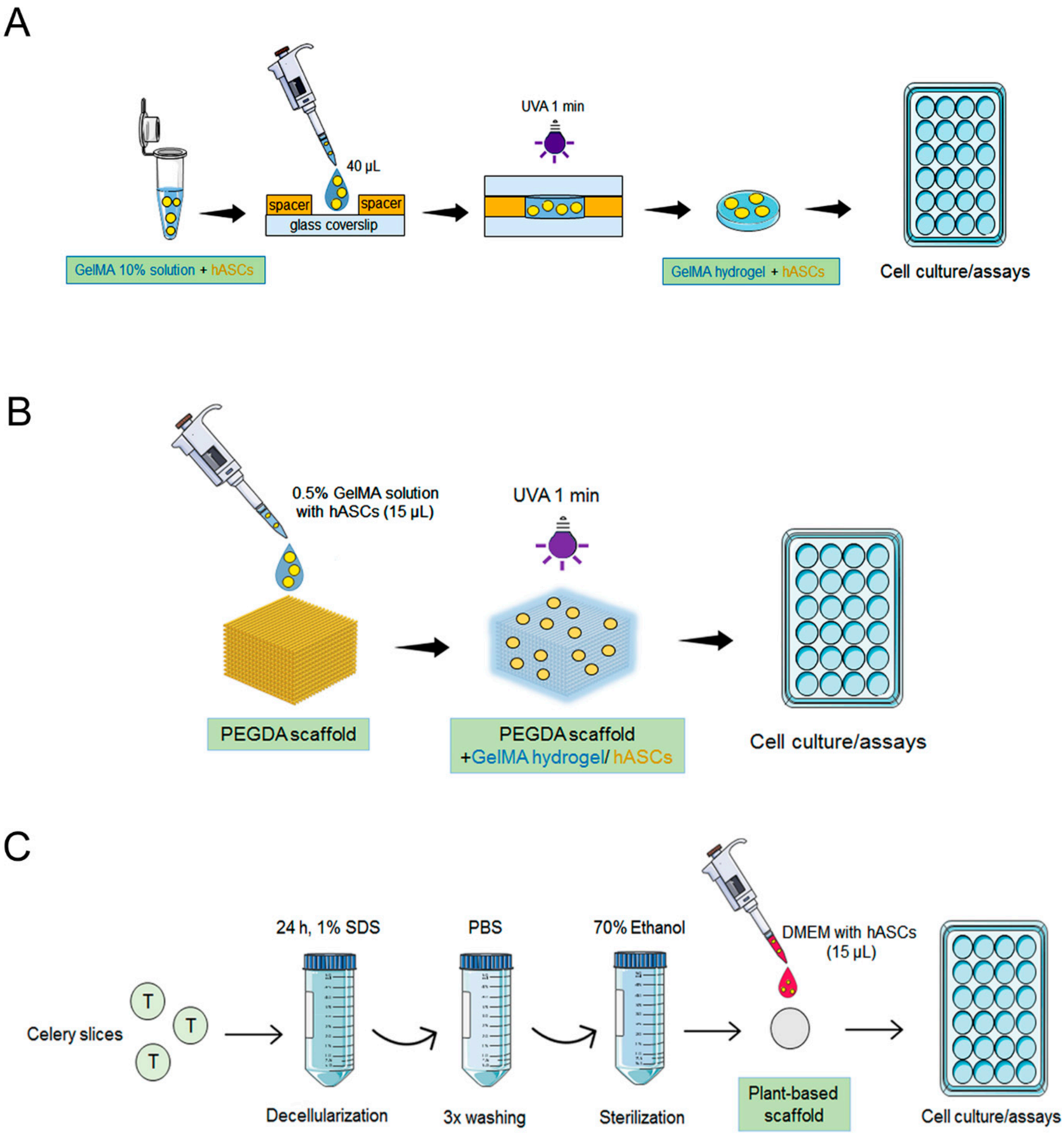

Figure S2. Scaffold cell seeding. (**A**) Cell seeding process, hASC suspension (300,000 cells/15µL solution) is mixed to 10% GelMA solution and placed on a sterile cover slip that is exposed to UV light for 1 minute for photo-crosslinking. Finally, GelMA hydrogel is placed in a non-adherent 24 multi-well plate and cultured with DMEM plus 10% FBS and kept in the incubator. (**B**) Encapsulation process of hASCs in PEGDA scaffold. A drop of 0.5% GelMA solution containing hASCs (300,000 cells/ 15µl solution) is pipetted out on PEGDA scaffold and exposed to 1 minute to UV light. Finally, the obtained scaffolds are placed in a non-adherent 24 multi-well plate and cultured with DMEM plus 10% FBS and kept in the incubator. (**C**) Cell seeding in celery-based scaffolds (transversal cut, 24h SDS protocol). After making and sterilizing the scaffold, a drop of hASC suspension (200,000 cells/15µl medium) is pipetted out on the top and let it rest for 2h in the incubator. Subsequently, scaffolds are placed in a non-adherent 24 multi-well plate and cultured with DMEM plus 10% FBS.

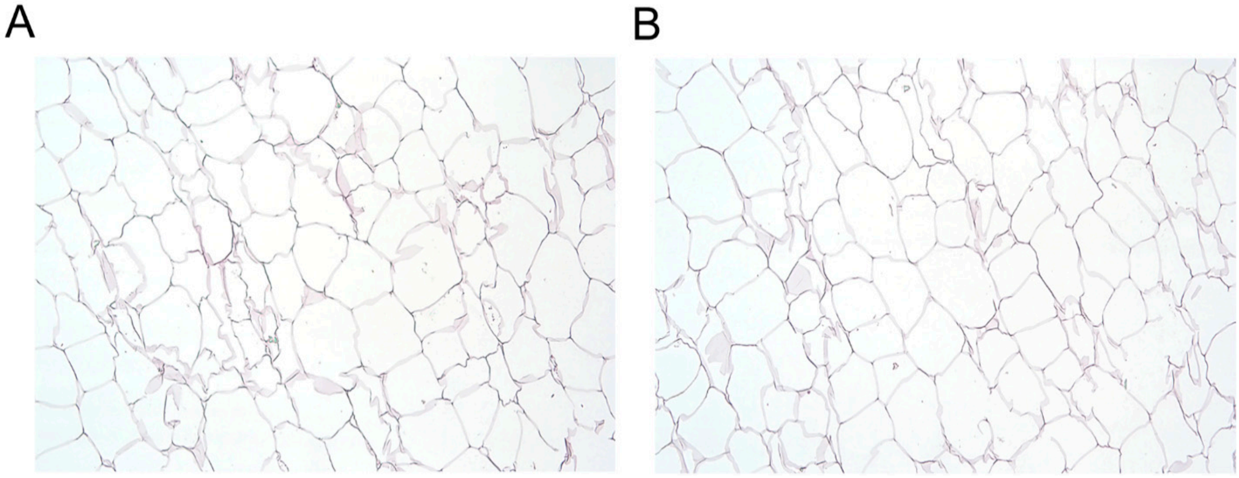

Figure S3. Plant base decellularization. (**A**) Plant-based scaffold transversal cut decellularized after 24h SDS protocol and (**B**) plant-based scaffold longitudinal cut decellularized after 24h SDS protocol.
